# Development and Modification of New or Recycled Materials and Technological Processes towards Sustainable Development

**DOI:** 10.3390/ma17133060

**Published:** 2024-06-21

**Authors:** Pavol Liptai

**Affiliations:** Institute of Recycling Technologies, Faculty of Materials, Metallurgy and Recycling, Technical University of Košice, Letná 9, 042 00 Kosice, Slovakia; pavol.liptai@tuke.sk

Quality and environmental sustainability are fundamental to the healthy and proper functioning of society on Earth. The current human population should want to leave the environment in a better condition or in the best possible condition for future generations. Therefore, each person must contribute to this goal, regardless of origin, race, religion, or social and occupational status. Then, this social consensus can be achieved. Sustainability should be the overriding and common goal of human civilization, whatever the context and how we approach it.

Many scientists and researchers are trying to develop various recycling technologies to recover high-quality raw materials, while others are working on ways to reduce or prevent waste or developing technologies to eliminate polluted water, soil, or air. Many are looking for ways to optimize technological processes to minimize their environmental impact or to ensure that the outputs of these processes are products that will be efficiently recycled at the end of their life cycle. However, it should be noted that these efforts will be meaningful only if everyone contributes and does not rely on others to solve the problems. Often, it is enough to realize that natural resources provide us with inputs for product manufacturing, and, at the same time, products at the end of their life cycle constitute a supply of materials for further use. This way, we understand the circular economy and environmental sustainability.

In this Special Issue titled “Development and Modification of New or Recycled Materials and Technological Processes towards Sustainable Development”, 16 papers were published that address specific tasks and problems to achieve long-term sustainability.

The five review papers address promising issues in various scientific fields. Fu et al. [1] describe advances in the development of layered double hydroxide-based materials for wastewater treatment applications. Takacova et al. [2] provide an overview of the current state of the field in spent portable lithium battery recycling at both the research and industrial scales. Another interesting review is offered by Pan et al. [3], focusing on the applications of luminescent silver nanoclusters embedded in zeolites for lighting, gas monitoring, and sensing. Mironovs et al. [4] concentrate their review on sustainable methods of recycling, using, or reusing perforated metal materials, and Rostami-Tapeh-Esmaeil and Rodrigue [5] provide a review based on the latest research on the morphological, physical, and mechanical properties of rubber foams, their applications, and future development.

The eleven research articles offer promising studies from various scientific fields with potential practical applications. Bottom zinc dross, as a valuable secondary material, is investigated in the paper by Pauerová et al. [6]. The recycling of cable waste and its application use are explored by Wędrychowicz et al. [7]. The improvement in electrocatalysts, based on a NiCoP surface for bifunctional water splitting, is examined by Sheng et al. [8]. The development of high-temperature in situ water sorbents, based on zeolite, dolomite, lanthanum oxide, and coke, is covered by Acha et al. [9]. The research by Pikna et al. [10] aims at optimizing the processing and recovery of chromium through the electrocoagulation method. The study by Mamrilla et al. [11] provides valuable insights into zinc-based alloys containing magnesium, calcium, and manganese, which can be used to design alloys for specific biomedical applications. Research on h-BN and g-C3N4 nanoparticles as oil-based additives for significantly reducing wear rates and thereby extending product life is presented by Zhong et al. [12]. The authors of Takacova et al. [13] focus on optimizing the process of impurity removal to obtain high-purity ZnO. The optimization of process parameters in induction hardening is investigated by Dziatkiewicz et al. [14]. The potential usability of recycled titanium scrap as a substitute for primary sources is explored in the work by Judd et al. [15]. Finally, the paper by Hejazi et al. [16] significantly enhances knowledge of the processes of the restoration and conservation of Persian historical buildings.
